# Management of hemolytic transfusion reactions in a patient with chronic myelomonocytic leukemia and rare antibodies: A case report

**DOI:** 10.1016/j.lrr.2024.100485

**Published:** 2024-11-28

**Authors:** Grace S. Park, Himachandana Atluri, Courtney D. DiNardo, Bryan Guillroy, Jean Horak, Effrosyni Apostolidou, Maryam Buni, Guillermo Montalban Bravo, Naveen Pemmaraju

**Affiliations:** a Department of Pharmacy, The University of Texas MD Anderson Cancer Center, Houston, TX, United States; b Department of Leukemia, The University of Texas MD Anderson Cancer Center, Houston, TX, United States; c Department of Laboratory Medicine, The University of Texas MD Anderson Cancer Center, Houston, TX, United States; d Department of Benign Hematology, The University of Texas MD Anderson Cancer Center, Houston, TX, United States; e Department of Rheumatology, The University of Texas MD Anderson Cancer Center, Houston, TX, United States

**Keywords:** Hemolytic transfusion reactions, CMML, Induction therapy

## Abstract

Delayed hemolytic transfusion reaction (DHTR) poses a significant challenge in patients receiving blood transfusions. This case report highlights the complexities of managing DHTR in a newly diagnosed chronic myelomonocytic leukemia (CMML) patient with clinically significant JKa and little c antibodies during induction chemotherapy. A 46-year-old woman with CMML-2 who presented for induction chemotherapy was found to have hemolytic anemia. Due to presence of JKa and little c antibodies, she required intensive monitoring and supportive care measures. The coexistence of JKa and little c antibodies complicates transfusion management and chemotherapy tolerance in CMML patients.

Delayed hemolytic transfusion reaction (DHTR) or delayed serologic transfusion reaction reflects the immune-mediated destruction of red blood cells (RBCs) due to the presence of antibodies that target blood cells or transfused blood cells. Alloantibodies, such as those targeting blood group antigens such as Kidd (JK blood group system - anti-JKa and anti-JKb) form in JKa and JKb negative individuals following pregnancy or RBC transfusions. The levels of these antibodies then drop precipitously following the next several months and below the level of detection upon subsequent RBC cross-matching. Thus, a ‘cross-match compatible’ RBC transfusion may trigger anamnestic response of such antibodies which will eventually lead to hemolytic reactions [1]. Sensitization to JKa or JKb antigens can occur through pregnancies or prior transfusions that express the corresponding antigens [2]. Additionally, antibodies against the little c antigen, part of the Rh blood group system, can also cause similar reactions resulting in hemolytic anemia. Proper blood typing and cross-matching are crucial to prevent these complications and ensure safe transfusions [3].

The coexistence of JK antibodies and little c antibodies in patients undergoing routine blood transfusions raises pertinent concerns regarding transfusion-related complications. Chronic myelomonocytic leukemia (CMML) is a complex hematologic malignancy characterized by dysregulated monocytic proliferation and dysplastic hematopoiesis. Cytopenia often arises from ineffective hematopoiesis and increases the need for blood transfusions. While the involvement of JK antibodies and little c antibodies in hemolytic transfusion reactions is recognized in various contexts, their specific impact on leukemia patients receiving active treatment is not well characterized [2,4]. Herein, we report the case of a patient with newly diagnosed CMML and the impact of clinically significant JK antibodies and little c antibodies during induction chemotherapy administration.

A 46-year-old Hispanic woman with a past medical history of hypertension, five successful pregnancies, and anemia (most pronounced in the post-partum setting of her youngest son in 2017) was referred to our hospital in March 2024 for newly diagnosed leukemia. She reported progressive fatigue, lightheadedness, and chest palpitation for 2–3 weeks prior to presenting at the local hospital. Initial labs were notable for WBC 20K, Hb 4.8, Plt 77K. Type and screen using Grifols Erytra analyzer (gel) platform revealeda positive antibody screen and ABORh type discrepancy. Anti-c or a little c antibody was identified. Type was repeated in tube using Quotient(Albacyte) Anti-A, Anti-B, Anti-D, A cells and B cells and compared to ABO type using a segment from Group A red cell unit negative for the little c antigen to resolve the type discrepancy. She received 3 units of packed red blood cells at the local hospital two days prior to presenting at our hospital with appropriate response in her hemoglobin to 8.3 g/dL. A bone marrow biopsy demonstrated: 15 % blasts with chronic myelomonocytic leukemia - 2 (CMML-2) with *FLT3, DNMT3A*, and *KMT2A* mutations. FLT3 ratio for D835 was 0.03 and ITD ratio was 0.01. She was started on intermediate-intensity treatment with cladribine 5 mg/m^2^ for 5 days, subcutaneous cytarabine 20 mg twice daily for 10 days, and gilteritinib 80 mg daily for 28 days. On day 2 of chemotherapy, her hemoglobin was 7.0 g/dL and she received 1 unit of blood. On day 4 of chemotherapy, her hemoglobin dropped from 8.1 g/dL to 5.4 g/dL without any signs of active bleeding and other symptoms. Her type and screen at this time revealed the presence of the JKa and little c antibody with undetectable haptoglobin, absolute retic was 0.0404 M/uL, LDH 545 (ULN 214) and direct antiglobulin test (DAT) positive consistent with delayed hemolytic anemia. Crossmatches were performed in the gel platform from Grifols, and positive DAT testing was investigated using Gamma ELU-KIT from Werfen. Benign hematology was consulted, and the patient was started on IVIG 2 g/kg divided in 4 days, weekly rituximab 375 mg/m2 IV, cyclosporine 200 mg twice daily, and high dose methylprednisolone 1 mg/kg for 5 days followed by weekly taper. The patient's family members were screened along with a nation-wide search for matched blood products. After 2 days, patient's hemoglobin eventually fell to a nadir of 4.5 g/dL, and total bilirubin increased from 0.3 mg/dL to 1.8 mg/dL. Her brother was found to have fully matched phenotype and donated 1 unit. The blood bank was able to procure four additional units of blood products for the patient over the following week. One of the units the patient received initially was positive for the Jka antigen, and all other units transfused were negative for both the little c and JKa antigens. Absolute retic fell to 0.0125 M/uL. Eltrombopag was also added to assist hematologic recovery based on literature in aplastic anemia showing multi lineage improvement in addition to folic acid, and epoetin alfa. Cyclosporine was discontinued due to elevated liver enzyme on day 5. During the first 14 days of chemotherapy, patient received 4 units of compatible blood products as well as 2 units of fully matched blood products. During the remainder of the induction therapy, she received 2 more units of compatible blood products, and her hemoglobin stayed between 5 and 7 g/dL (Fig. 1).

**Fig. 1:**
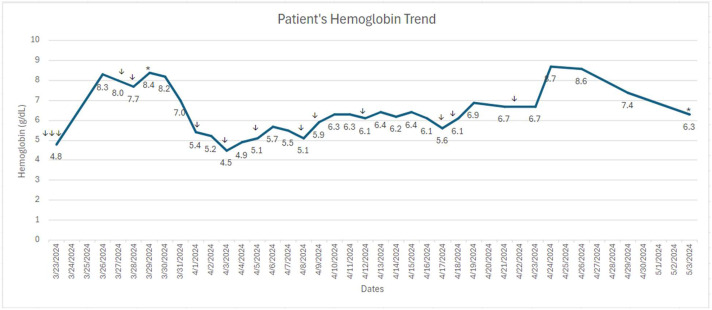
↓ Transfusion of each unit of red blood cell product. * Initiation of chemotherapy cycle.

Monitoring for disseminated intravascular coagulation (DIC) secondary to delayed hemolytic transfusion reaction and supportive care with cryoprecipitate with a goal of fibrinogen greater than 150 was also initiated, and pediatric tubes to minimize blood loss from routine blood draws were utilized. Labs were monitored every other day to minimize blood draws starting day 21 once hemoglobin stabilized. Due to DHTR and elevated liver enzymes, her chemotherapy regimen was attenuated to cladribine 5 mg/m2 for 4 days, subcutaneous cytarabine 20 mg twice daily for 5 days, and gilteritinib 80 mg daily for 4 days. Bone marrow biopsy on cycle 1 day 28 of her attenuated induction therapy revealed persistent CMML with 6 % blasts with FLT3 ratio 0.11 for D835 and ITD ratio <0.01. Patient's hemoglobin at day 28 was 8.6 g/dL with platelet 128 K/uL after 1 unit of matched red blood cell transfusion on day 27.

A week after discharge, the patient received a reinduction cycle with cladribine for 5 days, low dose cytarabine for 10 days, and gilteritinib 80 mg daily with her local oncologist. During the 10 days of admission for re-induction, she received 3 additional units of irradiated blood products and did not show any signs of hemolysis. She achieved a morphologic remission and she is planned for a curative allogeneic stem cell transplant.

JKa antibodies are alloantibodies that target the Jka antigen on RBCs and can lead to delayed hemolytic transfusion reactions when transfused with crossmatch incompatible blood. Treatment of delayed hemolytic anemia in patients with JKa antibodies and little c antigen involves a combination of supportive measures and immunosuppressive therapy. This may include (1) transfusion support with compatible blood products (2) immunosuppressive therapy to suppress immune response against RBC antigens with prednisone or methylprednisolone 1–2 mg/kg/day (3) Intravenous immunoglobulin (IVIG) to block Fc receptors on macrophages and inhibit antibody-mediated hemolysis (4) rituximab to deplete antibody-producing B cells and modulate immune response (5) splenectomy who failed previous lines of therapy to remove the site of RBC destruction and/or (6) close monitoring on hemoglobin levels, reticulocyte counts, markers of hemolysis such as lactate dehydrogenase or bilirubin to detect treatment response [[5], [6], [7], [8], [9]]. In patients with CMML or other hematologic malignancies, treatment of delayed hemolytic anemia due to alloantibodies may have additional nuances: (1) chemotherapy tolerance, (2) transfusion dependence, and (3) immune-related adverse events.

Chemotherapeutic agents used in CMML treatment, such as hypomethylating agents (e.g., azacitidine, decitabine) and cytotoxic drugs (e.g. cladribine), may exacerbate hemolytic anemia or trigger autoimmune responses in patients with pre-existing JKa antibodies. Particularly, cladribine containing regimen has shown to have immunomodulatory effect, which might have contributed to the immune-mediated reaction due to antigen exposure [12]. Individualized treatment strategies, including dose adjustments, treatment interruptions, or alternative therapies, may be necessary to manage hemolytic complications and optimize treatment outcomes in CMML patients with concurrent autoimmune hemolytic anemia and alloantibodies. In this case, the combination regimen of cladribine, low dose cytarabine, and gilteritinib was used for high risk CMML, particularly due to FLT3 mutations.

Overall, leukemia induction therapy causes universal myelosuppression, especially when it is combined with chemotherapy and a targeted agent [10,11]. Close monitoring of hematologic parameters, including hemoglobin levels, is essential during treatment to promptly identify and manage anemia and other hematologic toxicities. Supportive care measures, such as red blood cell transfusions and erythropoiesis-stimulating agents, should be employed to alleviate symptoms and maintain hemoglobin levels within an acceptable range.

Patients who develop alloantibodies with severe hemolytic anemia may become transfusion-dependent, requiring frequent red blood cell transfusions to maintain hemoglobin levels and alleviate symptoms. The presence of JKa antibodies complicates transfusion management, as compatible blood products must be carefully selected to avoid hemolytic reactions and alloimmunization. Close monitoring of hemoglobin levels, reticulocyte counts, and markers of hemolysis is essential to guide transfusion decisions in CMML patients with hemolytic anemia. Communication with the blood bank, including Jka-negative RBC units, is critical to prevent transfusion reactions and further alloimmunization. Screening family members can also help identify compatible blood products, especially in individuals with multiple antibodies.

Novel treatment modalities for CMML, including immunomodulatory agents and targeted therapies, may exacerbate autoimmune hemolysis or induce immune-related adverse events. Corticosteroids, immunosuppressants (e.g., cyclophosphamide, rituximab), and intravenous immunoglobulins (IVIG) may be employed to suppress autoimmune responses and alleviate hemolytic complications in CMML patients with JKa antibodies. In many cases of CMML, especially in those with severe hemolytic anemia or refractory disease, allogeneic HSCT may offer curative potential by replacing the dysfunctional hematopoietic system with a healthy donor graft. Pre-transplant evaluation for JKa antibodies and careful selection of compatible donors are essential to minimize the risk of graft rejection and immune-mediated complications post-transplant.

In summary, the presence of Kidd and Rh alloantibodies and hemolytic anemia can pose significant challenges in the treatment of CMML and other hematologic malignancies, impacting chemotherapy tolerance, transfusion management, and treatment outcomes. A comprehensive understanding of the underlying mechanisms and tailored management strategies are crucial to address these complications effectively and improve the quality of life for patients with alloantibodies.

## CRediT authorship contribution statement

**Grace S. Park:** Writing – review & editing, Writing – original draft, Visualization, Investigation, Data curation. **Himachandana Atluri:** Writing – review & editing, Investigation. **Courtney D. DiNardo:** Writing – review & editing, Supervision. **Bryan Guillroy:** Writing – review & editing, Supervision. **Jean Horak:** Writing – review & editing. **Effrosyni Apostolidou:** Writing – review & editing. **Maryam Buni:** Writing – review & editing. **Guillermo Montalban Bravo:** Writing – review & editing. **Naveen Pemmaraju:** Writing – review & editing, Supervision.

## Declaration of competing interest

None.

## Appendix Supplementary materials

Image, application 1
