# Supplementary material for: Management of hemolytic transfusion reactions in a patient with chronic myelomonocytic leukemia and rare antibodies: A case report
Source: Leuk Res Rep. 2024 Nov 28;23:100485. doi: 10.1016/j.lrr.2024.100485 (PMC11647623; doi:10.1016/j.lrr.2024.100485)
Supplement: Supplementary file 1 [file mmc1.pdf]

Media Information

Scan on 4/12/2024: Authorization for the Use and Disclosure of Protected Health Information - 2 of 2 pages printed

[redacted]

THE UNIVERSITY OF TEXAS
MD Anderson Cancer Center
Making Cancer History

Authorization for the Use and Disclosure of Protected Health Information – Media Use

Patient: [redacted]
MDA #: [redacted]
Date: 4/12/24
Sex: Female
DOB: [redacted]
FC: I

A copy of this form must be given to the signing individual.

**Any media requests (including, but not limited to, local, regional or national newspapers or magazines – online or print, television and radio stations, social media, Internet sites) must be approved and managed by the Communications Office at 713-792-0655.*

***Any advertising and marketing requests (including, but not limited to print media, radio or television, Internet sites, billboards, collateral material or promotional items) must be approved and managed by the Marketing Office at 713-792-1909.*

I authorize The University of Texas **MD Anderson Cancer Center and its employees, agents and representatives (MD Anderson)** to use or share my Protected Health Information (PHI), including photographs, videotaped images, audio/digital recordings and other images (Images) with:
The public
Name of organization/person

I allow MD Anderson to use or share the PHI that I indicate below:
Name
Demographic information (such as hometown, state)
Treatment History
Images (i.e., photo, video, audio recordings)
Diagnosis (specify):
Other (specify):

I allow PHI to be used or shared for these reasons:
Educational purposes, including presentation at a conference, forum, workshop or seminar
Other purposes (specify):

I understand that the information released may contain information related to AIDS or HIV infection, drug or alcohol abuse, mental or behavioral health or psychiatric care (other than psychotherapy notes) and authorize release of such information.

I understand this authorization will expire the later of one (1) year from the date the authorization is signed or upon the following date or event *(specify)*:
Case report to be written by the end of 2024

[redacted]

I understand that once disclosed, my information may be re-disclosed by the recipient and may no longer be protected by federal privacy regulations. **I may revoke this authorization** in writing at any time, except when MD Anderson has already relied on this authorization or the information is no longer under MD Anderson's control. I can revoke this authorization by sending a written request by mail to Privacy Officer, MD Anderson Cancer Center, Institutional Compliance Office, Unit 1640, PO Box 301407, Houston, TX 77230-1407, by fax to 713-563-424, or by email to privacy@mdanderson.org.

This authorization is **optional** and **I do not have to sign it**. Refusing to sign will not affect my treatment or payment for services.

PATIENT OR PERSONAL REPRESENTATIVE:
Is this consent being signed by the patient? YES

Patient Signature

04/12/2024 at 13:03:29 CDT

Is this consent being interpreted for the patient or the patient's personal representative? No

Document Information

Clinical Consent: General Consent

Authorization for the Use and Disclosure of Protected Health Information -

04/12/2024 00:00

Attached To:

Hospital Encounter on 3/26/24

Source Information

Interface, Onbase Scanning Incoming
